# Racism as a Social Determinant of Health in Brazil in the COVID-19 Pandemic and Beyond

**DOI:** 10.1055/s-0043-1770135

**Published:** 2023-06-20

**Authors:** Amanda Dantas-Silva, Silvia Maria Santiago, Fernanda Garanhani Surita

**Affiliations:** 1 Universidade Estadual de Campinas, São Paulo, SP, Brazil

The World Health Organization (WHO) defines *disparity* as the unnecessary, avoidable, and unfair treatment of two groups based on identified differences. 1 *Racial disparities* refer to the different treatment of distinct subgroups of people based on differences without any scientifically proven biological reason. 2

Growing evidence indicates that ethnic and racial disparities permeate health-related issues, and structural racism is key in determining population health. 3

Racism is a system of domination of a racial group defined as inferior by dominant groups that use phenotypic characteristics to justify inequalities in access to resources and power. 4 Racism is structural insofar as the social structure is constructed racially hierarchical. And racism is cultural in that the values and cultural beliefs of the dominant racial group are used as the norms by which other groups are socially judged. 5 Institutional racism, in turn, refers to the maintenance of racial inequalities by institutional mechanisms. This type of racism acts diffusively, as it is implicit in the daily functioning of institutions and social organizations through discriminatory practices that disadvantage certain people from accessing services and opportunities according to their skin color. 6 Thus, cultural racism maintains structural racism and both constitute the root of racial inequalities in health. 4 5

Health outcomes are directly impacted by the level of structural and institutional racism. 7 Racism is associated with worse physical and mental health outcomes, including increased depression, anxiety, and psychological stress based on the existence of racist beliefs and practices among health professionals about minority groups that influence their decision-making process and the care they provide. 8 9

Differences based on skin color permeate several areas of health. For instance, Black people receive fewer prescriptions of analgesics in general, less palliative treatment for metastatic cancer, and lowered use of revascularization therapy to treat cerebrovascular accidents (CVA). 10 11 12 Overall, people of color have worse cardiovascular outcomes and experience longer waiting times for care at the emergency department. 13 14

In Gynecology and Obstetrics, racial disparities are evident: Black women have the highest mortality rates and severe maternal morbidity, later onset and lack of prenatal care, are at higher risk of preeclampsia, prematurity, and postpartum hemorrhage, and report worse experiences during prenatal, delivery and postnatal care than other women. 15 16 17 18 19 20 21 Moreover, Black women have less access to contraceptive methods, receive more diagnoses of the human immunodeficiency virus (HIV) infection, 22 undergo fewer screening tests for cervical cancer, and have increased mortality rates from breast cancer 23 comparing to non-Black ones. 22 23 24

In Brazil, the majority of the population is Black ( *Negra* ). The Brazilian Institute of Geography and Statistics (IBGE) conceptualizes Black as people who self-declare as Black ( *Preta* ) and Brown ( *Parda* ). 25 Despite their majority, racial disparities are perpetuated against the Black population. 26

The COVID-19 pandemic exacerbated health inequalities. 27 The global public health emergency has imposed a new reality on health systems around the world and has accentuated inequalities in access to health services. Underdeveloped and developing countries were most affected by the effects of the pandemic, as existing socioeconomic inequalities affected the initial course of the disease and resulted in increased deaths from COVID-19, especially among the most vulnerable populations. 27 28 29

In Brazil, infection and death rates from COVID-19 are uneven, with greater risk among Black people and those with low socioeconomic status. 30 Mortality from COVID-19 was higher among the Black population, and maternal mortality was twice as high among Black women compared to all other women in our country. 31 32

The impact of the pandemic highlighted the existing racial disparities in health in Brazil. 33 An integrative review of Brazilian studies with population-based databases found that being Black was a risk factor independently associated with the severity of COVID-19. The authors concluded that the Black population suffered more than any other from the physical and economic impacts of COVID-19. 34

Racial disparities are evident in our population and marked sociodemographic differences remain between Black and non-Black women. In this issue of RBGO, a retrospective multicenter Brazilian study carried out with pregnant and puerperal symptomatic women suspected of having COVID-19 showed that Black women were younger and had less education, a higher rate of unplanned pregnancy, and greater public health insurance coverage. In addition, the findings showed greater severity of infection among Black women, with a higher risk of severe acute respiratory syndrome, admission to the Intensive Care Unit, greater desaturation on admission, and higher maternal mortality in this group. 35

Although the COVID-19 pandemic did not create these inequities, it reminded us of how structural racism is a driving force of the social determinants of health and highlighted the need for health professionals to change their approach and assistance, especially to discriminated populations. It means that all knowledge already produced about the health of the Black population should be appropriated by health professionals to deal more specifically with individuals, as well as call for greater production of knowledge about them. This is an important action to combat racism.

COVID-19 was not a democratic disease and further exposed the strong association between race, ethnicity, culture, socioeconomic status, and health outcomes. 36 Individual implicit prejudice and the profound impact of structural racism must be recognized and accepted before real progress can be made to reducing racial disparities in maternal mortality. To reduce the impacts of COVID-19 and other public health emergencies, it is urgent to adopt new models of care centered on women that consider racial disparities and overlapping vulnerabilities and develop public policies specifically aimed at the Black population while respecting their particularities.

Health inequalities are generated and maintained by social differences and unequal access to services, resources, and power. 1 Social determinants of health are non-medical conditions that involve the conditions in which people live, work, and grow and impact their risk factors and health outcomes. 37 38 These social determinants are responsible for health inequalities between countries and within the same country: generally, the worse the socioeconomic condition, the worse the health conditions. 1 37

The United Nation's sustainable development goals (SDG) for the years 2015 to 2030 include combating social disparities, of which we highlight three: SDG3 refers to health and well-being for all, SDG5 strives for gender equality, and SDG10 focuses on reducing inequalities. 39 Indeed, the COVID-19 pandemic had a strong impact on the goals to be achieved during this period, a delay of decades in several sectors, such as the reduction of maternal mortality, a health indicator strongly linked to our specialty, obstetrical gynecology. 40

Black women suffer from gender, social, and racial vulnerabilities that intersect and generate additive or multiplicative effects. 27 They also are under the impact of determinants produced by a historical movement, which built specific cultural ways of thinking about the Black population, as well as being vulnerable to the social conditions produced by an unequal society that affects their health. 41 The articulation of social determinants of difference was first thought by the Brazilian professor, philosopher, and author Lélia Gonzalez in the 80s, even before the intersectionality concept emerged. 42 Gonzalez relates the social makers of skin color, class, and gender in the racism construction and maintenance. The concept of intersectionality was then systematized by the American professor Kimberly Crenshaw as “the way in which racism, patriarchy, class oppression and other discriminatory systems create basic inequalities that structure the relative positions of women, races, ethnicities, classes, and others”. 43 Crenshaw proposes the concept as a method of locating inequalities suffered by Black women from structural racism. In this sense, Black women aggregate the largest set of unfavorable conditions and are placed at the bottom of the social pyramid. 44

Race is a social construction and researchers must consider the variable skin color within a historical context of discrimination as a complex variable that interferes with health outcomes not only due to genetic and biological factors but often due to social and economic factors. 45 46 Understanding racism and considering the existence of racial disparities in decision-making and the construction of public policies make it possible to reduce health inequalities.
